# Supplementary material for: Human seizures couple across spatial scales through travelling wave dynamics
Source: Nat Commun. 2017 Apr 4;8:14896. doi: 10.1038/ncomms14896 (PMC5382286; doi:10.1038/ncomms14896)
Supplement: Supplementary Information — Supplementary Figures, Supplementary Methods and Supplementary References [file ncomms14896-s1.pdf]

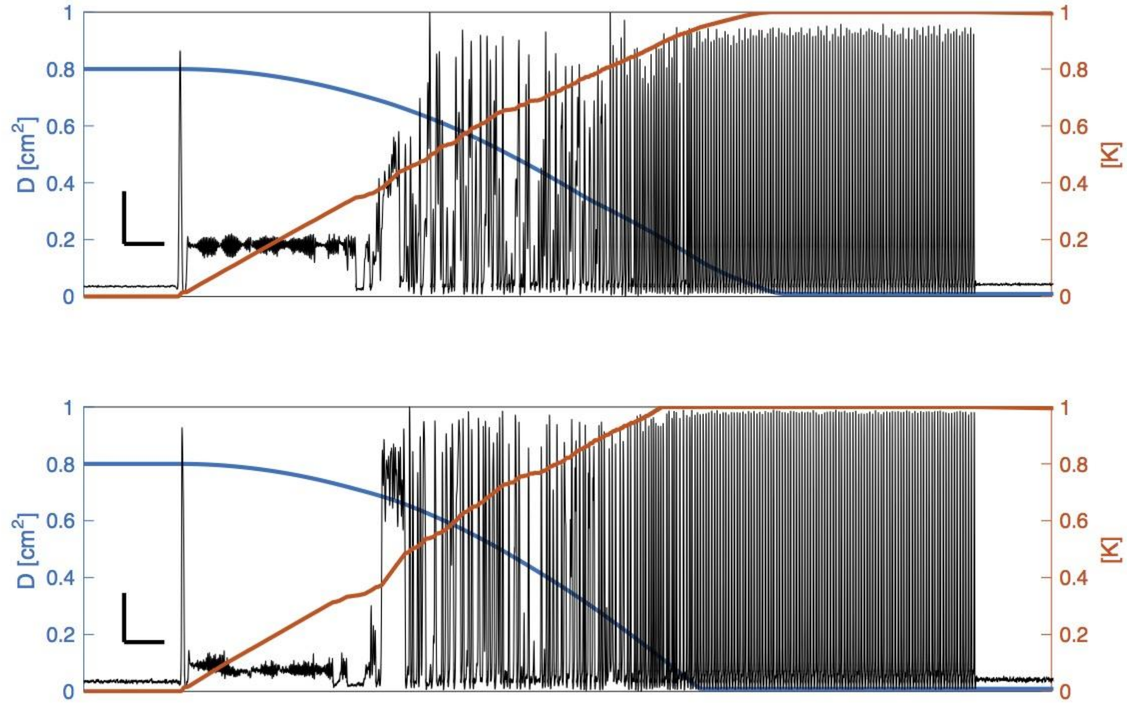

Supplementary Figure 1. The concentration of extracellular potassium increases, and the gap junction strength decreases, during simulated seizure. The voltage (black curve; vertical scale bar 5 Hz, horizontal scale bar 5 s), inhibitory-to-inhibitory gap junction strength (blue curve, left axis), and concentration of extracellular potassium (orange curve, right axis) at a macroelectrode (upper plot) and microelectrode (lower plot) during a simulated seizure. As the seizure evolves, the concentration of extracellular potassium increases and the inhibitory-to-inhibitory gap junction strength decreases. Both reach their extremum value approximately two-thirds of the way through the simulated seizure.

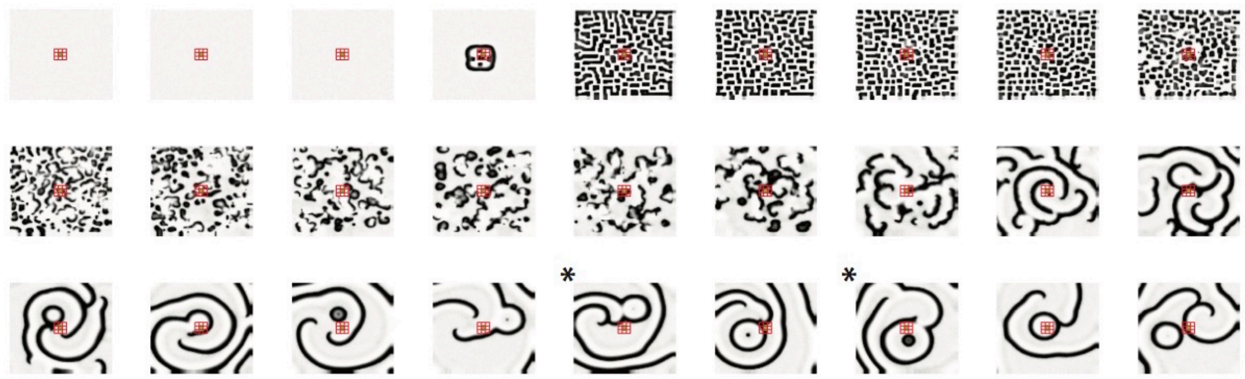

Supplementary Figure 2: Simulations of random source locations. Example spatial maps of simulated activity using random source locations. Each subfigure shows a snapshot of the excitatory population activity (white 0 Hz, black 25 Hz) on the 30 cm by 30 cm surface; the time between subfigures is 5 s, and time progresses from left to right, top to bottom. A cortical source, visible near the middle of the fourth subfigure, ignites the activity. Traveling waves are then driven by different seizure source locations which results in different wave patterns; an example of two different wave patterns are indicated by asterisks. See Supplementary Methods for more details. Simulated microelectrodes (green) and macroelectrodes (red) are indicated near the center of each map.

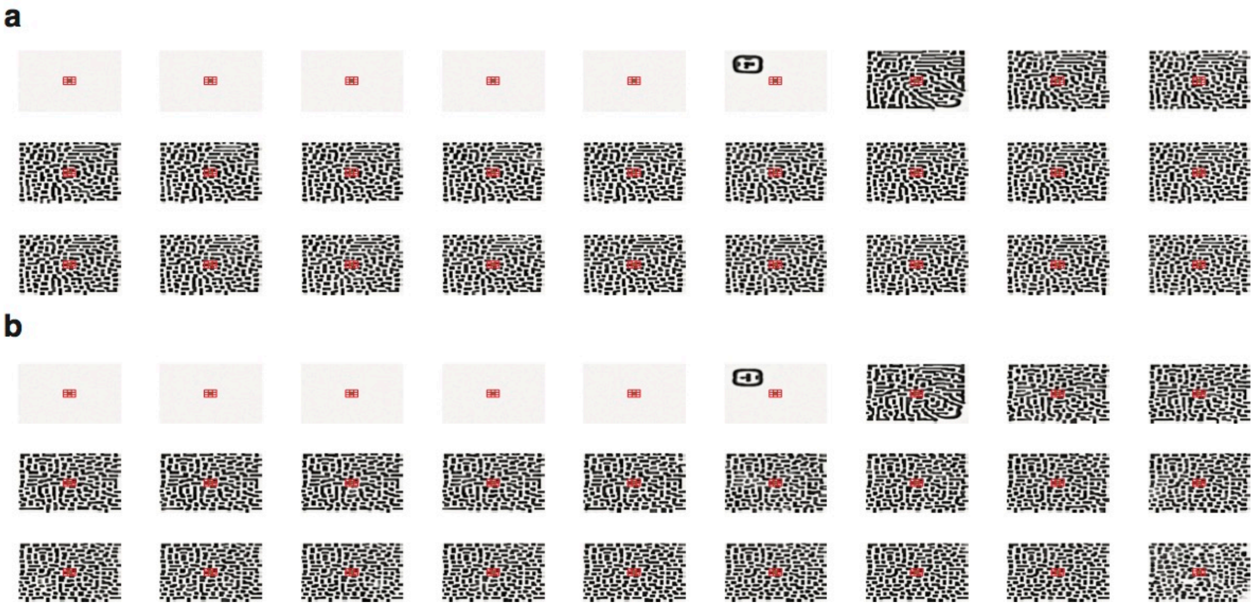

Supplementary Figure 3. Counteracting changes in the concentration of extracellular potassium or inhibitory-to-inhibitory gap junction strength prevents the transition to traveling waves. (a,b) Example spatial maps of simulated activity. Each subfigure shows a snapshot of the excitatory population activity (white 0 Hz, black 25 Hz) on the 30 cm by 30 cm surface; the time between subfigures is 5 s, and time progresses from left to right, top to bottom. The cortical source (visible near the upper left corner in the sixth subfigure) ignites the activity. After activation of the cortical source, (a) the concentration of extracellular potassium is returned to the pre-seizure value of 0, or (b) the inhibitory-to-inhibitory gap junction strength is returned to the pre-seizure value of 0.8, after each 1 s of simulation. These alterations prevent the slow changes in these variables that occur during the simulated seizure (example in Figure 5c). In both cases, mosaic patterns appear after source activation and these patterns continue for the entire duration of the simulation; traveling waves do not occur. In all figures the simulated microelectrodes (green) and macroelectrodes (red) are indicated near the center of each map.

Supplementary Methods

We consider two additional simulation scenarios in which we vary the spatial position of the cortical source of increased excitability. In the first scenario, the spatial location of the cortical source varies randomly in time (Supplementary Figure 2). The simulation begins with 40 s of background activity, without a source of increased excitability. Following this interval, the source is assigned a random 9 mm by 9 mm cortical location selected uniformly from the interior of the two-dimensional plane, between 75 mm and 225 mm in both the vertical and horizontal directions. The resting membrane voltages of the excitatory populations at the randomly selected source location are increased three-fold for a duration of 1 s. After this 1 s interval, the increased excitation at the selected location is removed, a new spatial location for the source is randomly selected, and the resting membrane voltages of the excitatory populations at this location are increased three-fold for a duration of 1 s. This process of randomly selecting each second a different spatial location for the source is repeated for 140 s. All of the simulation parameters match those used to create Figure 5; only the location of the source differs.

In the second simulation scenario (an example shown in Figure 6), we simulate the dynamics of an expanding ictal wavefront[1,2]. The simulation begins with 40 s of background activity, after which we increase three-fold the resting membrane voltage of the excitatory populations in a 9 mm by 9 mm cortical patch at position (120 mm, 120 mm); this patch defines the initial position of the ictal wavefront. The ictal wavefront then expands from this initial location in the following way. At each point on the boundary of the ictal wavefront, we identify all neighboring spatial locations that have not yet been invaded by the ictal wavefront. Then, for each point on the boundary, we randomly (uniformly) select one of these neighbors and advance the ictal wavefront to this neighboring location. After each point on the boundary has been advanced, we use the MATLAB function *boundary* with shrink factor 0.5 to determine a single conforming two-

dimensional boundary that encompasses all of the advanced points. The purpose of this last step is to define a boundary without any breaks; this boundary defines the ictal wavefront, which expands approximately radially over the two-dimensional cortical sheet[2]. At all points on the ictal wavefront the resting membrane voltages of the excitatory populations are increased three-fold. We set the ictal wavefront to advance at a speed of 1 mm/s[1]. In this simulation scenario, all parameters are identical to those used to create Figure 5, except that we set the offset to the resting potential of the excitatory population to -1 mV (versus +1 mV in Figure 5). The purpose of this change is to confine the traveling waves produced by the ictal wavefront to the territory encompassed by the ictal wavefront (i.e., the recruited region[2]).

Supplementary References

1. Schevon, C. A. *et al.* Evidence of an inhibitory restraint of seizure activity in humans. *Nat. Commun.* **3,** 1060 (2012).
2. Smith, E. *et al.* The ictal wavefront is the spatiotemporal source of discharges during spontaneous human seizures. *Nat. Commun.* **7 SP -,** (2016).
